# Adjoint-Driven Inverse Design of a Quad-Spectral Metasurface Router for RGB-NIR Sensing

**DOI:** 10.3390/nano15211671

**Published:** 2025-11-03

**Authors:** Rishad Arfin, Jeongwoo Son, Jens Niegemann, Dylan McGuire, Mohamed H. Bakr

**Affiliations:** 1 Department of Electrical & Computer Engineering, McMaster University, Hamilton, ON L8S 4K1, Canada; arfinr@mcmaster.ca; 2 Ansys Korea, 100, Toegye ro, Jung gu, Seoul 04631, South Korea; jeongwoo.son@ansys.com; 3 Ansys Canada Ltd., 1700-1095 West Pender Street, Vancouver, BC V6E 2M6, Canada; jens.niegemann@ansys.com (J.N.); dylan.mcguire@ansys.com (D.M.)

**Keywords:** color filters, inverse design, adjoint sensitivity, metasurface spectral router

## Abstract

There has been an increasing demand for high-resolution image sensing technologies in recent years due to their diverse and advanced optical applications. With recent advances in nanofabrication technologies, this can be achieved through the realization of high-density pixels. However, the development of high-density and miniaturized pixels introduces challenges to the conventional color filters, which generally transmit and absorb different spectral components of light. A significant portion of the incident light is inherently lost using conventional color filters. Moreover, as the pixel size is shrunk, optical losses appear to be substantial. To address these fundamental limitations, a novel nanophotonic optical router is proposed in this work. Our router utilizes a single-layer, all-dielectric metasurface as a spectral router. The metasurface is designed through an inverse design approach that exploits adjoint sensitivity analysis. A novel figure of merit is developed and incorporated in the inverse design process, enabling the metasurface design to effectively sort and route the incoming light into four targeted channels, each corresponding to a distinct spectral component—red, green, blue, and near-infrared. We demonstrate that the proposed quad-spectral metasurface router, having a compact footprint of 2 μm×2 μm, achieves an average optical efficiency of approximately 39% across the broad spectral range, i.e., 400–850 nm, with each spectral channel exceeding an efficiency of 25%. This surpasses the maximum efficiency attainable by the conventional four-channel color filters. Our proposed quad-spectral metasurface router offers a wide range of applications in low-light imaging, image fusion, computational photography, and computer vision. In addition, this work highlights the applicability of an adjoint-based inverse design approach to accelerate the development of compact, efficient, and high-performance nanophotonic devices for the next generation of imaging and sensing systems.

## 1. Introduction

The widespread demand and adoption of image sensors for various sensing applications [1,2,3] is largely driven by the rapid development of CMOS technology. Recently, advanced image sensors that capture images in both visible (i.e., red (R), green (G), and blue (B)) and near-infrared (NIR) regimes have emerged to provide additional information. This critical ability offers a wide range of applications, including computational imaging [4], surveillance [5,6], and sensing in low-light [7,8] and adverse environments [6,8]. However, high-resolution images appear to be fundamental for accurate detection, effective recognition, and enhanced performance of the system. In principle, high-resolution images require a large number of pixels per unit area. There are two approaches to accommodate high-density pixels. First, an increase in the surface area, without changing the pixel size, allows for an enhanced number of pixels. While this approach improves optical sensitivity, it leads to an enlarged device footprint, thus limiting its applicability for compact systems. Second, a reduced pixel size within a fixed surface area leads to a higher density of the pixels. This latter approach is widely adopted because it is compatible with current trends toward device miniaturization. However, reducing pixel size introduces two major challenges. First, as the pixel size shrinks, the collection of light for each pixel reduces, resulting in significant degradation in image quality [9,10]. Second, as the pixel size reduces, the conventional microlenses become less efficient at focusing due to strong diffraction of the incoming light [11]. Consequently, unintended spectral light is collected by the adjacent pixels, compromising the image quality.

Several approaches [12,13,14,15] have been employed to develop efficient designs of microlenses for enhanced optical performance in conventional image sensors. However, these design strategies explore a limited design space, such as by varying the geometric profiles, positions, and radii of curvature of microlenses. A systematic and efficient microlens shape optimization technique for enhanced focusing was studied in [16], enabling access to a larger design space. Nevertheless, the conventional absorptive color filters, used beneath the microlenses in image sensors, set a fundamental limit on optical efficiency. For instance, each pixel of the image sensor ideally receives only 25% of the incident light [17] when using a conventional RGB-NIR color filter.

An intuitive approach to overcome the limitations of conventional microlenses and absorptive color filters is to develop a nanophotonic device that efficiently routes incoming light across the structure to the targeted region. This functionality can be achieved using metasurfaces, as it is capable of manipulating light at subwavelength scales. Recently, metasurface spectral routers [17,18,19,20,21,22,23,24,25,26] have drawn considerable interest due to their ability to selectively control and redirect the light of specific wavelengths to designated pixels.

Different designs of metasurface routers have been proposed and investigated in several works [19,21,27,28,29,30] for enhanced performance. The studies presented in [21,28,29] have leveraged dielectric deflectors and pillars to effectively split the incoming light based on the spectral wavelengths. Symmetric and asymmetric geometries of deflectors are utilized in [28] to induce wavelength-dependent angular deflection. As the propagation phase delay depends on the geometry and refractive index of the material, different spatial orientations of small pillars across the surface were used in [21,30] to generate variation in the phase profiles. While designs based on angular deflection and phase modulation offer control over the direction and phase of light, these design approaches exhibit several drawbacks. First, the deflectors or pillars typically cover a very limited space in the unit cell, leaving a substantial amount of incident light unguided on the surface. This results in significant crosstalk. Second, a long propagation distance is often required in these structures to achieve adequate spatial separation of multiple wavelengths before reaching the target region [17]. This induces diffraction of light in the structure, leading to increased crosstalk. Third, designs based on phase modulation are often derived from a precomputed phase library or lookup table, which maps the required phase shifts to their corresponding nanostructure geometries. While constructing a detailed phase library is computationally exhaustive, using a limited set of geometries in the metasurface design results in discrete phase shifts. This compromises the design accuracy and overall performance. Furthermore, a phase library generated from a finite set of nanostructure geometries [21,30] inherently limits the exploration of a larger design space, potentially missing better solutions.

**Figure 1:**
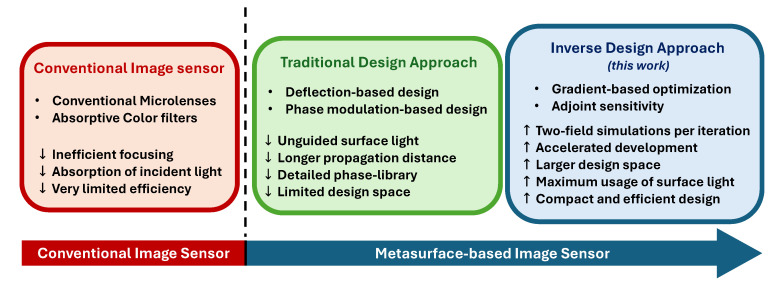
An overview of conventional and metasurface-based image sensors, showing a comparison between traditional and inverse design approaches for the metasurface router.

To address the shortcomings of traditional metasurface router design methodologies, we adopt a gradient-based inverse design approach to develop the metasurface spectral router. The gradients required to guide the optimization process are efficiently computed by leveraging adjoint sensitivity [31,32,33,34]. The advantages of the adjoint-based inverse design method are threefold. First, only two full-field electromagnetic (EM) simulations are required to compute the gradient of the figure of merit in each iteration [31], regardless of the number of optimizable parameters. This accelerates the systematic and efficient development of the metasurface router. Second, unlike traditional design methods, this approach utilizes the entire surface of the metasurface router. This ensures that all incident light on the surface is effectively guided to the target spectral channels. Furthermore, efficient utilization of the entire surface allows the optimizer to navigate through a larger design space, enabling it to determine highly efficient and compact yet nonintuitive metasurface configurations. Third, while the optimizer explores a vast parameter space, the computational overhead for gradient estimation using adjoint sensitivity remains insensitive to the number of design parameters. Figure 1 presents an overview of conventional image sensors and metasurface-based image sensors, alongside a contrast between traditional and inverse design approaches of metasurface spectral routers.

In this work, we present a single-layer, all-dielectric, quad-spectral metasurface router (QSMR), which sorts and routes the incident light into four different channels for RGB-NIR sensing. The adjoint-driven inverse design approach is used to develop the QSMR with a pixel size of 1 μm×1 μm. The proposed QSMR separates linearly polarized incident light into three visible wavelengths (RGB) and a near-infrared (NIR) channel. Dielectric materials are selectively used to construct the QSMR owing to their low optical loss [35]. The presented QSMR demonstrates optical efficiencies of 38.6%, 47.2%, 42%, and 29.3% for blue, green, red, and near-infrared wavelengths, respectively, surpassing the maximum efficiency of the conventional color filters. Moreover, an average efficiency of around 39% is calculated over the wideband, ranging from visible to near-infrared. The efficient and compact QSMR, presented in this work, is compared with the RGB-NIR metasurface routers previously reported [21,26,36]. Our proposed QSMR is well-suited for diverse applications, including low-visibility detection, environmental monitoring, and remote sensing.

The rest of this paper is organized as follows: In Section 2, we discuss the computational inverse design methodology of the QSMR, highlighting the novel figure of merit (FOM), followed by the adjoint-based gradient estimation process. The results are presented, analyzed, and discussed in Section 3. Section 4 summarizes the study with a concluding remark.

## 2. Inverse Design Methodology

**Figure 2:**
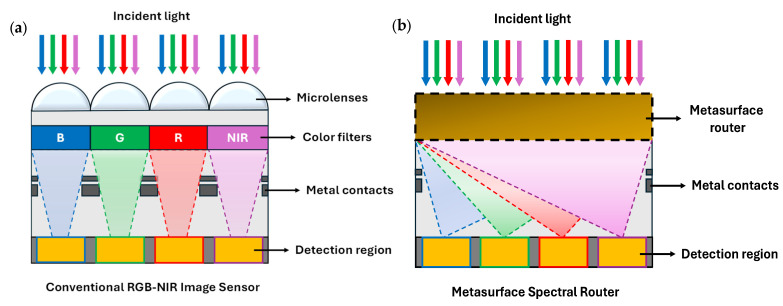
A schematic of (**a**) a conventional RGB-NIR image sensor with traditional microlenses and color filters, and (**b**) a metasurface router for RGB-NIR sensing.

A conventional RGB-NIR image sensor includes microlenses and color filters placed above the detection region, as shown in Figure 2a. While the microlenses focus the incident light, the color filters perform wavelength-dependent transmission or absorption. For instance, a red color filter selectively transmits only 25% of the incident light in an ideal condition. Thus, a substantial amount of incident light is wasted across a unit cell, having mosaic-like pixels. Alternatively, the unified function of focusing and wavelength-dependent optical transmission can be carried out by a metasurface router. The metasurface router simultaneously performs separation and spatial routing based on the wavelength, redirecting the incident light to the target region, as illustrated in Figure 2b. Unlike traditional design approaches presented in [21,28,29,30], our proposed QSMR effectively utilizes the entire surface to manipulate the incident light. Utilizing the entire surface of the structure as a design region allows the exploration of a larger design space.

To efficiently explore and navigate through a vast design space, we employ a gradient-based optimization approach. In this method, an efficient search is carried out in the design space to determine the design parameters that optimize (or maximize) the FOM. The gradient of the FOM is computed to guide the optimization process. There are several exhaustive approaches [37] to calculate the gradient of the FOM. In this work, we use adjoint sensitivity [31,32,33,34] to accurately compute the gradient of the FOM with respect to the design parameters. The adjoint method performs gradient computation with minimal computational overhead, requiring only two field simulations irrespective of the number of design parameters. Once the gradient is computed, the optimization algorithm uses the gradient information to improve the FOM by modifying the design parameters of the metasurface router. This design modification process is iterative. The process stops once a predefined termination condition, such as convergence, is satisfied or the design reaches a threshold-binarization, achieving the target functionality. The advantages of using gradient-based algorithms in nanophotonic inverse design are twofold. First, as the photonic design space is typically nonconvex [38], the gradient-based optimization method can identify multiple distinct designs (i.e., local optima), achieving target functionalities. Second, while the most optimized (or best) design may not be feasible to fabricate, the gradient-driven method, with different initializations and constraints, may produce several good local designs [39].

**Figure 3:**
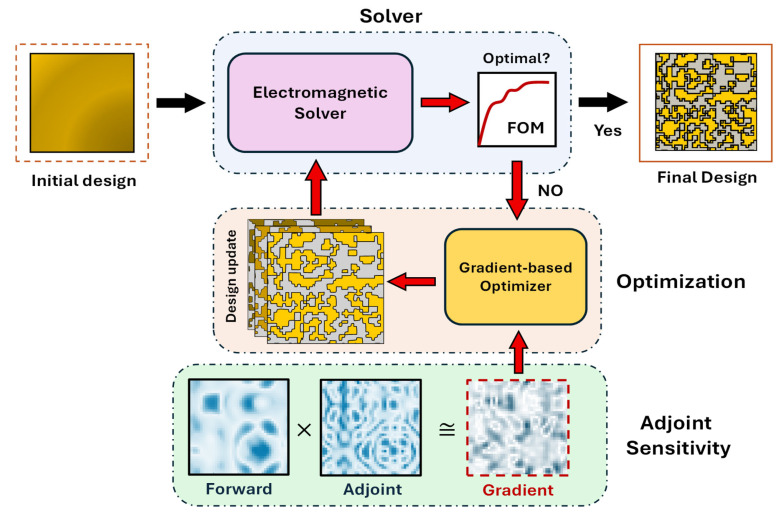
An illustration of the inverse design methodology of a metasurface spectral router using adjoint sensitivity.

The computational methodology used to develop the QSMR in this work is depicted in Figure 3. This mainly includes an electromagnetic (EM) solver and a gradient-based optimizer. The EM solver evaluates the FOM of the QSMR, followed by an optimization algorithm that iteratively adjusts the design parameters of the QSMR based on FOM sensitivity. In each iteration, the required FOM sensitivities with respect to all design parameters are efficiently calculated using adjoint sensitivity. The optimization process halts once the QSMR achieves the target functionalities. In the following subsections, we briefly discuss the structure and materials used to develop QSMR, followed by the predefined FOM. Moreover, the gradient-based optimization method is presented, highlighting the adjoint sensitivity for gradient computation. Furthermore, we discuss the performance metric used in this work to evaluate the proposed QSMR design.

### 2.1. Structural Modeling

In this study, the numerical implementation of the metasurface structure is carried out using a finite-difference time-domain (FDTD)-based EM solver. We use Ansys Lumerical FDTD [40] to account for the strong diffraction effects arising from the reduced pixel size. Moreover, the time domain simulation performed using FDTD inherently captures the wideband response, allowing the evaluation of the optical performance of the proposed QSMR.

**Figure 4:**
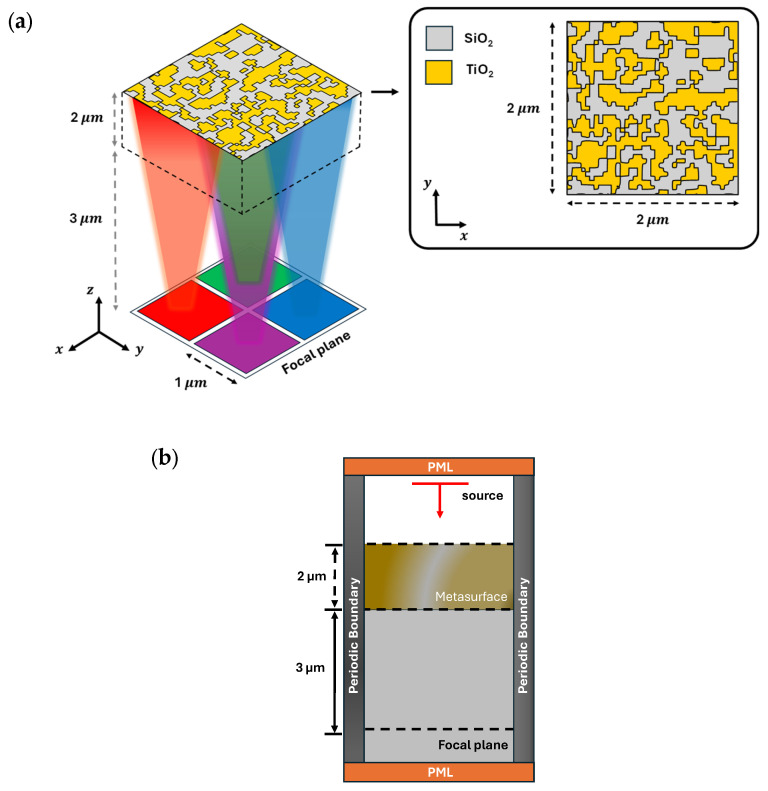
(**a**) A schematic of the QSMR, illustrating the efficient binary distribution within the unit cell of size 2 µm × 2 µm during in-plane optimization. (**b**) A schematic of the simulation setup highlighting absorbing and periodic boundary conditions.

The RGB-NIR image sensor architecture consists of a 3D metasurface structure placed on a glass substrate. The size of the metasurface is 2 µm × 2 µm × 2 µm, as presented in Figure 4. The design region of the proposed metasurface includes design cells as design parameters, each having a subwavelength size of 50 nm × 50 nm. While we carry out in-plane (i.e., xy-plane) optimization of the metasurface, as shown in Figure 4a, the thickness of the structure (i.e., along the vertical z-axis) is kept constant to ensure fabrication feasibility. We opt for a thickness of 2 µm for the metasurface to ensure sufficient wavelength-dependent phase accumulation as the light transmits through the metasurface. Dielectric materials TiO_2_ and SiO_2_, having constant permittivity values of 5.76 and 2.1, respectively, are used for design cells. During optimization, the gradient of the FOM is iteratively computed using adjoint sensitivity with respect to 1681 design parameters, where each design cell is expected to take a permittivity value (i.e., 5.76 or 2.1). While the optimization initially allows design cells with intermediate permittivity values, between 5.76 and 2.1, a binarization process is employed during the later phases of the optimization, gradually pushing the intermediate permittivity values toward the two discrete bounds (i.e., 5.76 or 2.1). This results in an efficient and manufacturable binary structure consisting only of TiO_2_ and SiO_2_ across the metasurface. The distance of the focal plane from the metasurface structure is set to 3 µm to achieve a compact QSMR configuration. In this study, we use TM-polarized normal illumination to excite the QSMR. Perfectly matched layers (PMLs) and periodic boundary conditions are applied to the metasurface unit cell, as depicted in Figure 4b, representing absorbing boundary conditions and periodicity, respectively.

### 2.2. Figure of Merit (FOM)

In this study, we define an FOM that reflects the ability of the structure to efficiently focus the spectral light at targeted regions during the optimization process. The light intensity at the center of each channel is defined as a part of the FOM, analogous to the one presented in our previous work [16]. The overall FOM of the proposed QSMR to simultaneously route the light in four spectral channels is given by the following:

(1) $$\mathrm{FOM}=\sum_{i=1,2,3,4}{\mathrm{FOM}}_{i}=\sum_{i=1,2,3,4}w_{i}{\left|E(r_{i},\lambda_{i})\right|}^{2},$$

where i=1, 2, 3, and 4 correspond to blue, green, red, and near-infrared channels, respectively. In Equation (1), E denotes the electric field at the focal point $r_{i}$ of the $i^{\mathrm{th}}$ wavelength-channel, and $\lambda_{i}$ indicates the central wavelengths for blue, green, red, and near-infrared (450 nm, 550 nm, 650 nm, and 750 nm, respectively). The parameters $w_{i}$ represent the optimization weights, ranging from 0 to 1, assigned to each wavelength. The simultaneous optimization of each spectral FOM may conflict inadvertently. Thus, the appropriate selection of asymmetric weights can help to achieve a balanced performance across multiple wavelengths.

### 2.3. Gradient-Based Optimization

In this study, we use the limited-memory Broyden–Fletcher–Goldfarb–Shanno (L-BFGS) optimization algorithm [41] to maximize the overall FOM. In each iteration, the optimization method searches for an optimal set of design parameters in the design space that improves the overall FOM. This search technique is guided by sensitivity information and strongly depends on the initial conditions (or design) of the QSMR structure. The L-BFGS uses first-order sensitivity, i.e., the gradient, and efficiently approximates second-order sensitivity, i.e., the inverse Hessian, to determine the search direction [42]. This implies that the optimization method used is strongly driven by the gradient of the FOM. Once a search direction is determined, an iterative line search is carried out along the direction of the gradient to calculate the optimal step size. This enables the optimizer to perturb the design parameters of the QSMR, thus improving the overall FOM. The process of adjusting the design parameters for improved FOM repeats until a target FOM is achieved or a termination condition (e.g., the structure becomes sufficiently binarized, or shows negligible improvement in the FOM over successive iterations) is satisfied.

### 2.4. Adjoint Sensitivity

**Figure 5:**
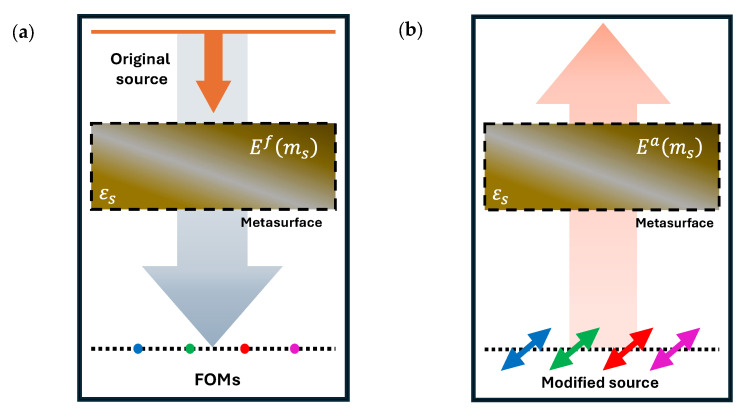
An illustration of the calculations of adjoint sensitivities: (**a**) the forward field is determined in the optimization domain with position vectors ***m****_s_*, and (**b**) the adjoint field is also determined in the same optimization domain to compute the FOM sensitivities of the proposed QSMR for RGB-NIR sensing.

While the L-BFGS optimization method primarily relies on the gradient of the FOM to adjust the design parameters, it efficiently approximates the inverse Hessian based on the changes in the gradient and design parameters. Therefore, accurate estimation of the gradient is imperative while navigating a photonic design space with a large dimensionality. In this work, we leverage adjoint sensitivity to calculate the gradient of the FOM efficiently. Adjoint sensitivity computes the gradient of the FOM with respect to all the design parameters using only two field simulations—forward (or physical field) simulation and adjoint (or nonphysical field) simulation, as shown in Figure 5. The detailed formulation of the adjoint field for the optimization of nanophotonic devices is presented in [43,44,45].

In this work, we developed a novel FOM and implemented an FDTD-based adjoint formulation to approximate the gradient of the FOM corresponding to the $i^{\mathrm{th}}$ channel as follows:

(2) dFOMidεs≅Re[Efms·Eams],

where $E^{f}\left(m_{s}\right)$ and $E^{a}\left(m_{s}\right)$ indicate the phasors of the forward and adjoint electric fields, respectively, at each point $m_{s}$ within the QSMR, having a dielectric permittivity of $\varepsilon_{s}$. The gradient of the FOM corresponding to the $i^{\mathrm{th}}$ channel of the metasurface, expressed in Equation (2), is calculated in every iteration, as shown in Figure 3, to update the dielectric permittivity distribution of the QSMR for enhanced performance.

### 2.5. Optical Efficiency

We evaluate the performance of the developed QSMR to determine its ability to route the incident light across the structure to the respective spectral channels. Optical efficiency is used as a performance metric, representing the fraction of the incident optical power received by the targeted pixels across the spectrum. The optical efficiency for each spectral channel is given as follows [17]:

(3) $$\mathrm{Optical Efficiency}=\frac{P_{t}(i)}{P_{\mathrm{inc}}}$$

where i  corresponds to the spectral channels, $P_{t}(i)$ denotes the power transmitted at the targeted focal plane of the $i^{\mathrm{th}}$ channel, and $P_{\mathrm{inc}}$ reflects the source power. The Poynting vector over the intended surface at the targeted focal plane can be integrated to compute the power transmitted at each channel.

## 3. Results

**Figure 6:**
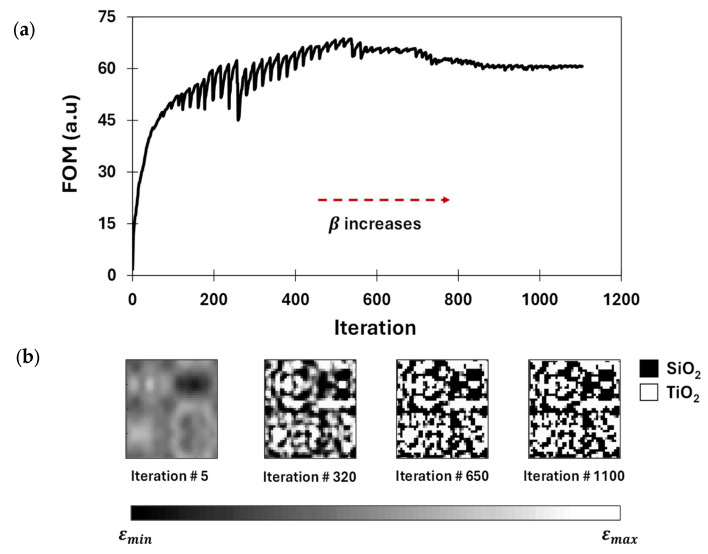
Illustration of the achieved results: (**a**) the progression of the FOM during the optimization process and (**b**) the permittivity distribution at different iterations, illustrating the structural transition of the metasurface.

In this section, we present the in-plane optimization results of a 3D metasurface with dimensions of 2 µm × 2 µm × 2 µm, demonstrating its ability to achieve the target functionality for RGB-NIR sensing. The gradual development of the FOM and QSMR design during the optimization process is illustrated in Figure 6. The progression of the FOM, presented in Figure 6a, reflects the optimizer’s attempts to find an optimal permittivity distribution in each iteration to improve the FOM. The gradient-based optimization starts with an initial design, where the permittivity distribution is uniformly set to the average of the dielectric permittivity of TiO_2_ and SiO_2_. The forward and adjoint field simulations are carried out using an EM solver in every iteration to compute the gradient. This helps the optimizer to iteratively update the permittivity value of each design cell (or design parameter) along the direction of the estimated gradient using a projection filter [46,47]. The projection filter is used to binarize the structure over the optimization process, making it physically realizable and compatible with the standard fabrication processes. The sharpness of the projection filter is regulated by the binarization factor β. Initially, the permittivity values represent fictitious material for small values of the binarization factor β, representing a non-realizable structure. Over the optimization, as the binarization factor β gradually increases, the projection filter becomes sharper, pushing the intermediate permittivity values toward the terminal values, i.e., 5.76 and 2.1. Consequently, this results in sharp drops in the FOM, followed by gradual FOM improvement over the next successive iterations, as shown in Figure 6a. This occurs because the optimizer adapts with the increased values of β and attempts to improve the FOM for an enhanced level of binarization. The optimization process continues until the structure is sufficiently binarized at large values of the binarization factor β, or the FOM indicates no further improvement in achieving target functionalities. While the FOM shows rapid improvement during the early steps of the optimization process, as illustrated in Figure 6a, there is negligible to no significant improvement in the FOM after around 850 iterations. Moreover, the FOM reaches almost a plateau after 1000 iterations. The gradual evolution of the permittivity distribution of the metasurface at different iterations is presented in Figure 6b, demonstrating the transition from grayscale to a sufficiently binarized structure. This suggests that our optimization process has reached convergence.

**Figure 7:**
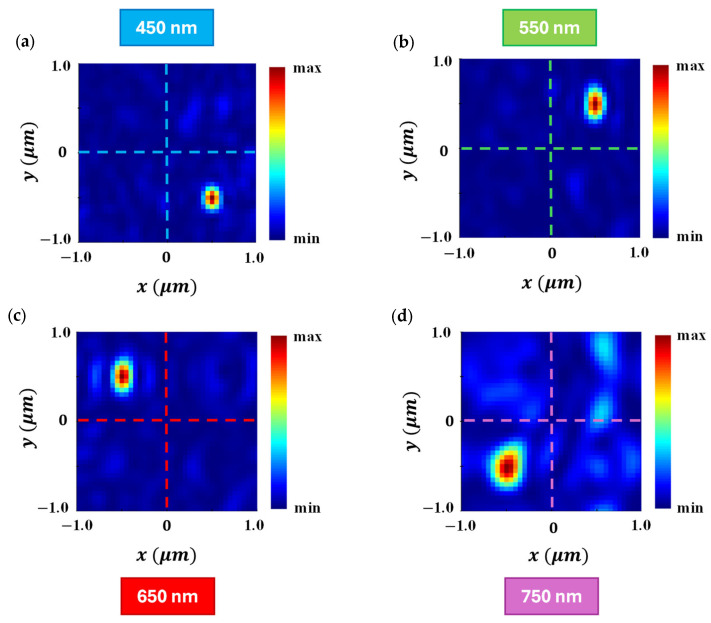
The distribution of the optical field at the focal plane for the proposed QSMR at wavelengths of (**a**) 450 nm, (**b**) 550 nm, (**c**) 650 nm, and (**d**) 750 nm.

The optical field distribution at the focal plane for the developed QSMR is illustrated in Figure 7. The enhanced optical intensity at the four targeted pixels, each measuring 1 μm×1 μm and corresponding to the four considered wavelengths, is shown in Figure 7a,b,c,d, respectively. The QSMR exhibits strong optical intensity at 450 nm, 550 nm, and 650 nm, as shown in Figure 7a,b,c, respectively, demonstrating effective isolation across the visible spectrum. While some portion of the optical power is distributed in the adjacent non-targeted pixels at 750 nm, a substantial amount of light is still strongly confined at the center of the targeted pixel, as presented in Figure 7d. This confirms that the developed QSMR can efficiently route and focus the incident light on four different spectral channels simultaneously.

**Figure 8:**
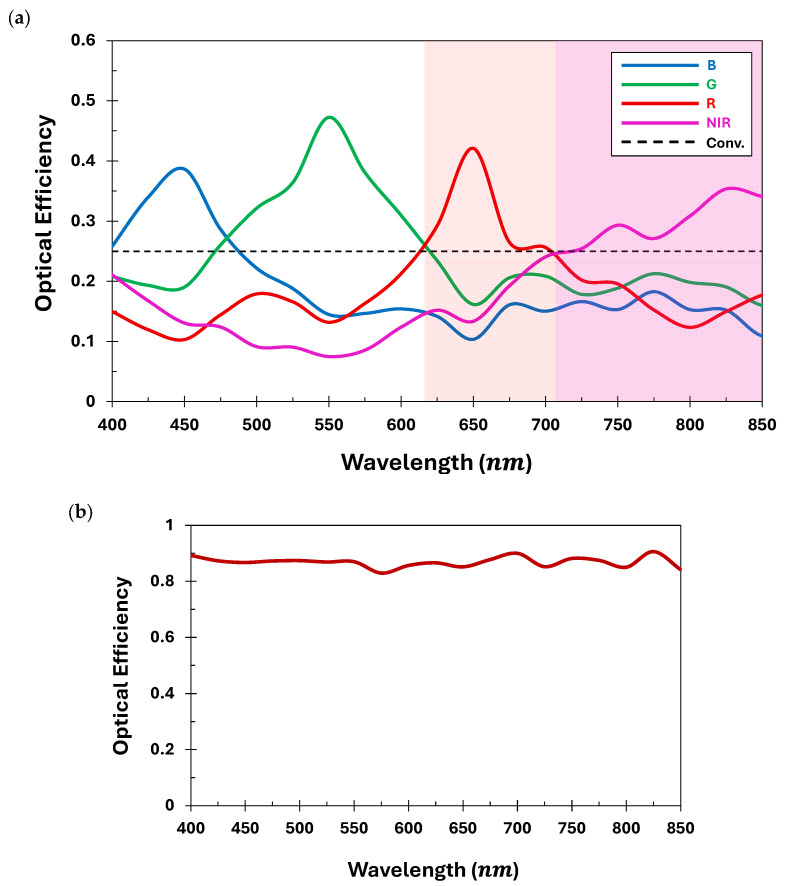
(**a**) The optical efficiency of the proposed QSMR (shown in solid lines) and conventional color filters (shown in dotted lines) over a wideband spectrum, ranging from visible to near-infrared, (**b**) the total optical efficiency at the focal plane of the proposed QSMR across the visible and near-infrared spectrum.

We estimate the optical efficiency of the developed QSMR over the wideband, i.e., 400 nm–850 nm, covering both visible and near-infrared. The ratio of the integrated power density over the targeted pixel area to the total incident optical power is evaluated to determine the optical efficiency, as presented in Equation (3). The optical efficiencies corresponding to the four different spectral channels are illustrated in Figure 8a. Figure 8a shows that each channel of the QSMR achieves optical efficiencies of 38.6%, 47.2%, 42%, and 29.3% at the target wavelengths of 450 nm, 550 nm, 650 nm, and 750 nm, respectively. Moreover, the average optical efficiency across the broadband is calculated to be approximately 39%. This significantly outperforms the conventional RGB-NIR absorptive-color filters, which can attain a maximum efficiency of 25%, as presented in Figure 8a. These results collectively suggest that the QSMR, developed using an adjoint-driven inverse design method, can simultaneously perform efficient routing of the incident light across the structure into four distinct spectral channels. Furthermore, the average total optical efficiency at the focal plane of the proposed QSMR reaches over 85% across the visible and near-infrared spectrum, as shown in Figure 8b.

**Figure 9:**
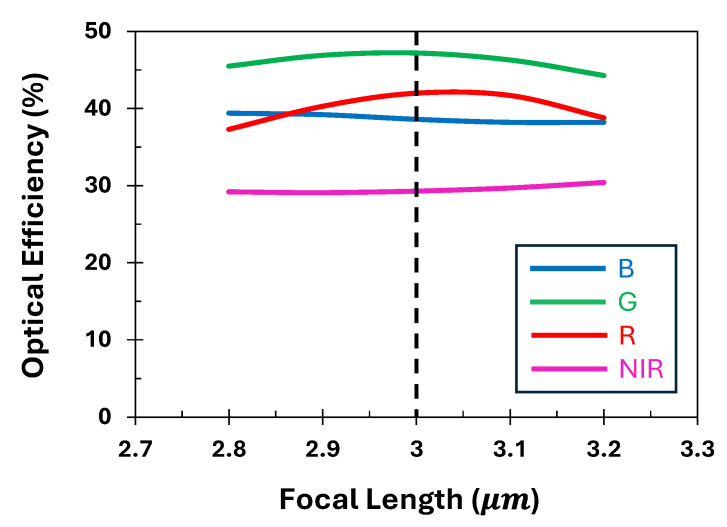
Optical efficiencies of the proposed QSMR across different channels with a variation of focal length.

Moreover, to account for the potential fabrication and measurement tolerances, we evaluate the sensitivity and robustness of the proposed QSMR for each channel with respect to variations in the focal length. Figure 9 presents the optical efficiencies of the adjoint-optimized QSMR across four different channels as the nominal focal length varies. While the optical efficiency of the targeted channel for the proposed QSMR exhibits relatively higher sensitivity to the focal length variation, the compact router overall demonstrates a robust performance across other channels.

In contrast to the previously reported metasurface routers, which are limited to two channels [30,48] and three channels [19,20,27,28,49], a key feature of our proposed QSMR is its ability to simultaneously route light across four distinct spectral channels. Our proposed inverse design methodology explores a larger photonic design space, unlike the traditional design approaches. Although the gradient-based search technique is sensitive to initial conditions, it identifies multiple high-performance designs that meet the target functionalities. This provides flexibility in design selection that is compatible with existing fabrication constraints. The adjoint-based inverse design methodology presented in this work is polarization-agnostic and can be adapted for TM, TE, or unpolarized conditions without loss of generality. To achieve a similar target functionality over a spectral band for unpolarized illumination would require FOM development for multi-objective optimization, which simultaneously considers both orthogonal polarizations. The FOM we developed in this approach can be further modified for non-targeted pixels through the inclusion of additional terms to mitigate the residual crosstalk. In addition, optimization weights can be adjusted and tuned to meet design specifications and desired functionalities. Further research could include an extension to the adjoint-based inverse design approach to capture the potential edge effects and evaluate the overall performance of a finite metasurface array in real-world scenarios. This can be achieved by incorporating a modified FOM during optimization to account for edge effects or by adding absorptive color filters at the edges only to mitigate the potential crosstalk. Moreover, the representative metasurface optical stack used in this study can be improved further by including additional layers and optical components, such as anti-reflection coating and metal wirings, within the inverse design framework in future studies.

Table 1 presents a comparative summary of four channels of metasurface routers, previously developed for RGB-NIR sensing. The design of the metasurface router in [21] consists of geometrical nanostructures generated from a phase library. Another study [36] used a genetic algorithm (GA) to develop an RGB-NIR metasurface router using an inverse design approach. The average optical efficiencies across the wideband spectral regime for the metasurface routers developed in [21] and [36] are slightly over 32% and 35%, respectively. In comparison, the QSMR presented in our work achieves an enhanced average efficiency of approximately 39% across the visible and near-infrared spectrum. While the metalens-based structure constructed in [26], through the combination of deep learning (DL) and particle swarm optimization (PSO) approach, achieves a slightly higher average efficiency of over 40%, our proposed QSMR design offers significantly more compact configuration, as shown in Table 1. The well-balanced combination of high optical efficiency and compact footprint makes our proposed QSMR suitable for next-generation sensing and imaging applications.

## 4. Conclusions

We presented a single-layer all-dielectric QSMR, demonstrating its ability to route light efficiently to the four different spectral channels for RGB-NIR sensing. The efficient design of QSMR is developed using an adjoint-based inverse design approach. The proposed QSMR exhibits an average optical efficiency of around 39% across a broad spectral band spanning the visible to near-infrared regime, with all spectral channels achieving significantly higher efficiencies over 25%. The presented QSMR design outperforms the maximum optical efficiency attainable with conventional color filters. Moreover, the optical performance and compact footprint of the QSMR are comparable to those of the existing metasurface color routers and reflect the state-of-the-art performance. Our proposed QSMR can be integrated with miniaturized RGB-NIR image sensors used for low-light imaging, image fusion, and high-resolution multispectral imaging applications in compact systems.

## Figures and Tables

**Table 1:** A comparison with the previous works on RGB-NIR metasurface routers.

Ref.	Design Configuration		Dielectric Material	Design Method	Optical Efficiency (%)			
	Focal Length	Pixel Size			B	G	R	NIR
[21]	5.15 μm	2 μm × 2 μm	Si_3_N_4_	Phase Modulation	26.5%	28.6%	37.1%	39.1%
[36]	2 μm	1 μm × 1 μm	Si_3_N_4_	GA + Inverse	29.0%	38.0%	39.0%	36.0%
[26]	4.6 μm	2 μm × 2 μm	Si_3_N_4_	DL + PSO	41.62%	40.51%	42.15%	40.50%
This work	3 μm	1 μm × 1 μm	TiO_2_, SiO_2_	Adjoint + Inverse	38.6%	47.2%	42.0%	29.3%

## Data Availability

The original contributions presented in this study are included in the article. Further inquiries can be directed to the corresponding author.
